# Genetic and Molecular Basis of Cleft Lip and Palate: A Comprehensive Review

**DOI:** 10.3390/diagnostics16142269

**Published:** 2026-07-20

**Authors:** Beste Kamiloglu, Mohammad Talal Radwan

**Affiliations:** Department of Orthodontics, Faculty of Dentistry, Near East University, Mersin 99138, Turkey; mohammadtalal.radwan@neu.edu.tr

**Keywords:** cleft lip and palate, craniofacial anomalies, genetics, gene–environment interaction, embryology, genome-wide association studies, precision medicine, developmental biology

## Abstract

Cleft lip and palate (CL/P) are among the most common congenital craniofacial anomalies, arising from disruptions in facial development during early embryogenesis. These conditions show significant clinical and genetic heterogeneity and are broadly classified into syndromic and nonsyndromic forms. The objective of this review is to summarize current knowledge on the embryological, genetic, and molecular mechanisms underlying CL/P and to highlight their clinical implications. A comprehensive review of the literature was conducted, focusing on studies in developmental biology, human genetics, and genomics related to CL/P. Emphasis was placed on both syndromic and nonsyndromic forms, including findings from genome-wide association studies, gene mutation analyses, and investigations of gene–environment interactions. Syndromic clefting is frequently associated with pathogenic variants in genes such as *IRF6*, *TP63*, and *TBX22*, which play key roles in epithelial differentiation, transcriptional regulation, and palatal development. In contrast, nonsyndromic CL/P results from complex interactions between multiple genetic variants and environmental factors. Genome-wide association studies have identified numerous susceptibility loci, many located in noncoding regulatory regions active during craniofacial development. Environmental influences, including maternal nutrition, smoking, alcohol exposure, and folate metabolism, have been shown to significantly modify risk. CL/P is a multifactorial condition involving intricate interactions between genetic and environmental factors. Advances in genomics and developmental biology have enhanced understanding of its etiology and are contributing to improved risk assessment, diagnosis, and the development of future precision medicine approaches.

## 1. Introduction

Cleft lip and palate (CL/P) are among the most common congenital craniofacial anomalies, affecting approximately 1 in 700 live births worldwide, although considerable variation exists among different populations and geographic regions [1]. The prevalence is highest in Asian and Indigenous populations and lowest in African populations, reflecting the combined influence of genetic susceptibility and population-specific environmental factors [1,2]. CL/P arise from incomplete fusion of facial structures during early embryogenesis and may affect the lip, the palate, or both. In addition to the characteristic craniofacial defect, affected individuals frequently experience feeding difficulties, speech and hearing impairments, dental anomalies, and psychosocial challenges, often requiring lifelong multidisciplinary management [3].

Although cleft lip and palate are traditionally classified into syndromic and nonsyndromic forms, this distinction is not absolute at the molecular level. Syndromic CL/P is generally caused by rare, highly penetrant pathogenic variants in single genes and occurs together with additional congenital anomalies, whereas nonsyndromic CL/P results from the combined effects of multiple common and rare genetic variants interacting with environmental factors. Nevertheless, increasing evidence demonstrates substantial genetic overlap between these two categories. Several genes, including *IRF6*, *TP63*, *GRHL3*, *MSX1*, and components of major developmental signaling pathways, have been implicated in both syndromic and nonsyndromic forms depending on the type, location, and functional consequences of the genetic variant.

Advances in genome-wide association studies, next-generation sequencing, and functional genomics have identified hundreds of genes and susceptibility loci associated with craniofacial development. However, the strength of evidence supporting individual genes varies considerably. While numerous loci have been reported, only a relatively small number have been consistently replicated across independent populations or functionally validated in experimental models. Consequently, distinguishing well-established disease genes from newly proposed or population-specific candidates remains a challenge for clinicians and researchers.

Therefore, rather than attempting to catalogue all reported genes, this review focuses on genes and molecular pathways with the strongest and most reproducible genetic, functional, and clinical evidence. By integrating embryological mechanisms, developmental signaling pathways, gene–environment interactions, and recent genomic discoveries, this review aims to provide a clinically relevant synthesis of current knowledge while highlighting the shared and distinct molecular mechanisms underlying syndromic and nonsyndromic cleft lip and palate.

The etiology of CL/P is complex and multifactorial. Although early studies focused primarily on environmental causes, such as maternal illness or nutritional deficiencies, extensive genetic research has established that hereditary factors play a central role in disease susceptibility. Familial aggregation studies demonstrate a substantially increased recurrence risk among first-degree relatives, while twin studies consistently report higher concordance rates in monozygotic than dizygotic twins, supporting a strong genetic contribution alongside environmental influences [4,5]. Clinically, CL/P are classified as syndromic or nonsyndromic. Syndromic CL/P occur as part of defined genetic syndromes and are typically caused by highly penetrant pathogenic variants in single genes. In contrast, nonsyndromic CL/P account for approximately 70% of cases and result from complex interactions between multiple genetic variants and environmental exposures [6]. This distinction is important because it influences genetic testing strategies, recurrence risk assessment, and clinical management.

Normal lip and palate formation is a highly coordinated developmental process that occurs between the fourth and tenth weeks of gestation. Fusion of the medial nasal and maxillary prominences forms the upper lip, whereas the secondary palate develops through growth, elevation, and fusion of paired palatal shelves [7]. These events require tightly regulated neural crest cell migration, epithelial–mesenchymal interactions, cellular proliferation, apoptosis, and extracellular matrix remodeling. Disruption of any of these processes may lead to cleft formation [7,8].

Recent advances in molecular genetics have substantially improved understanding of the biological mechanisms underlying CL/P. Numerous genes encoding transcription factors, signaling molecules, and structural proteins have been implicated in craniofacial development, particularly within the *SHH*, *TGF-β*, *WNT*, *BMP*, and *FGF* signaling pathways [8,9]. Furthermore, genome-wide association studies (GWAS) and next-generation sequencing have identified numerous susceptibility loci and rare pathogenic variants, many of which are located within noncoding regulatory regions that influence gene expression rather than protein structure [10,11,12]. Environmental factors, including maternal smoking, alcohol consumption, folate deficiency, diabetes, and teratogenic medications, further modify disease risk through complex gene–environment interactions [11].

Despite these advances, the genetic architecture of CL/P remains incompletely understood. While numerous susceptibility genes and signaling pathways have been identified, their functional interactions and clinical significance continue to be elucidated. In addition, the rapid expansion of genomic technologies has generated new insights that are not comprehensively integrated within existing reviews. Therefore, an updated synthesis of current evidence is needed to provide a cohesive understanding of the molecular and genetic mechanisms underlying CL/P.

Particular emphasis is placed on the overlap and differences between syndromic and nonsyndromic CL/P, the rationale for focusing on the most strongly validated susceptibility genes, and the emerging molecular mechanisms that are shaping current concepts of cleft pathogenesis and precision medicine.

## 2. Materials and Methods

A narrative literature review was conducted using the PubMed, Scopus, and Web of Science databases. The literature search included studies published in English up to March 2026 using combinations of the following keywords: cleft lip, cleft palate, orofacial clefts, genetics, gene mutations, craniofacial development, molecular pathways, neural crest cells, genome-wide association studies, and gene–environment interactions. Original research articles, systematic reviews, meta-analyses, and landmark studies relevant to the genetic basis and pathogenesis of cleft lip and palate were included. Studies not published in English, conference abstracts, editorials, and articles not directly related to the scope of this review were excluded. Priority was given to recent publications while retaining seminal studies essential for understanding the field.

## 3. Embryology and Developmental Biology of Cleft Lip and Palate

The development of the human face is a highly coordinated and complex process that occurs primarily between the fourth and tenth weeks of embryonic life. During this critical window, precise spatial and temporal regulation of cellular migration, proliferation, differentiation, and apoptosis is required to ensure normal formation of the lip and palate. Disruption of any of these events—whether due to genetic mutations or environmental insults—can result in cleft lip, cleft palate, or combined CL/P [13].

### 3.1. Early Craniofacial Development and Facial Prominences

Facial morphogenesis begins around the fourth week of gestation with the formation of five facial prominences: one frontonasal prominence, paired maxillary prominences, and paired mandibular prominences. These structures are largely populated by cranial neural crest cells, which migrate from the dorsal neural tube into the developing facial region [14]. Neural crest cells are pluripotent and give rise to much of the craniofacial skeleton, connective tissue, and peripheral nervous system, making their proper migration and differentiation essential for normal facial development.

Early craniofacial morphogenesis is orchestrated by tightly regulated molecular signaling networks that govern neural crest cell specification, migration, proliferation, and differentiation. Sonic hedgehog (SHH) signaling establishes the craniofacial midline and promotes facial prominence outgrowth, whereas WNT signaling regulates neural crest cell induction and survival. Fibroblast growth factor (FGF) and bone morphogenetic protein (BMP) pathways coordinate proliferation and patterning of the facial mesenchyme, while transforming growth factor-β (TGF-β) signaling contributes to epithelial–mesenchymal communication required for normal facial morphogenesis. Crosstalk among these pathways ensures the spatial and temporal coordination necessary for normal lip and palate formation, and disruption of their activity can lead to cleft development [14,15].

The upper lip and primary palate arise from fusion of the medial nasal prominences with the maxillary prominences. Failure of this fusion process results in cleft lip with or without involvement of the primary palate. Genetic disturbances affecting neural crest cell survival, migration, or patterning during this stage can lead to unilateral or bilateral cleft lip [15].

### 3.2. Development of the Primary Palate

The primary palate forms anterior to the incisive foramen and originates from the merged medial nasal prominences. This structure contributes to the alveolar ridge and the premaxilla. Proper development of the primary palate requires tightly regulated epithelial adhesion and subsequent epithelial seam breakdown to allow mesenchymal continuity. Defects in epithelial differentiation or persistence of the epithelial seam can prevent complete fusion and result in clefting [16].

Fusion of the primary palate requires coordinated regulation of epithelial adhesion, apoptosis, and epithelial–mesenchymal transition. The transcription factors *IRF6*, *TP63*, and *GRHL3* regulate periderm differentiation and maintenance, preventing premature epithelial adhesions before fusion occurs. TGF-β signaling also contributes to epithelial seam remodeling, while interactions between epithelial and neural crest-derived mesenchymal cells coordinate tissue continuity. Dysregulation of these molecular processes results in persistence of the epithelial seam and failed fusion of the facial prominences [16].

Experimental studies have demonstrated that disruption of *IRF6*, *TP63*, or *GRHL3* impairs periderm differentiation, epithelial integrity, and medial edge epithelial remodeling, resulting in failed fusion of the primary palate. These findings highlight the essential role of epithelial regulatory networks in early craniofacial morphogenesis [17].

### 3.3. Secondary Palate Development

The secondary palate forms posterior to the incisive foramen and gives rise to the hard and soft palate. Its development begins around the sixth week of gestation and proceeds through several well-defined stages: palatal shelf outgrowth, elevation, midline contact, and fusion [7].

Initially, paired palatal shelves grow vertically on either side of the tongue. As mandibular growth and tongue descent occur, the palatal shelves elevate rapidly to a horizontal position above the tongue. This elevation is driven by intrinsic forces within the palatal mesenchyme, including changes in extracellular matrix composition and cellular reorganization [18].

Secondary palate development is regulated by coordinated molecular signaling between the oral epithelium and underlying mesenchyme. SHH signaling stimulates mesenchymal proliferation through epithelial–mesenchymal interactions, whereas BMP and FGF pathways regulate palatal shelf growth and patterning. TGF-β3 is essential for medial edge epithelial seam disintegration by controlling apoptosis, epithelial–mesenchymal transition, and cell migration. WNT signaling contributes to epithelial integrity and tissue patterning, while extracellular matrix remodeling facilitates shelf elevation and fusion. Together, these pathways form an integrated regulatory network that ensures successful palatogenesis [19,20].

Once elevated, the palatal shelves grow toward the midline, where their opposing medial edge epithelia establish contact and undergo fusion. Failure of these tightly regulated cellular and molecular processes results in cleft palate [18,19,20,21,22,23,24,25].

### 3.4. Distinct Developmental Mechanisms of Cleft Lip and Cleft Palate

Although often grouped together clinically, cleft lip and cleft palate have distinct embryological origins. Cleft lip arises from failure of fusion between facial prominences, whereas cleft palate results from defects in palatal shelf growth, elevation, or fusion. This distinction is supported by genetic studies demonstrating that certain genes are preferentially associated with cleft lip, while others are more strongly linked to isolated cleft palate [26].

Although cleft lip and cleft palate share several developmental pathways, their molecular regulation differs according to developmental timing and tissue-specific gene expression. Lip formation is more dependent on signaling pathways controlling facial prominence growth and fusion, including *SHH*, *FGF*, and *WNT* signaling, whereas secondary palate development relies heavily on TGF-β3-mediated epithelial seam disintegration, epithelial–mesenchymal interactions, and extracellular matrix remodeling. In addition, transcription factors such as *IRF6*, *TP63*, *TBX22*, *MSX1*, and *SATB2* exhibit distinct spatial and temporal expression patterns during craniofacial development, contributing to the phenotypic diversity of orofacial clefts. These molecular differences help explain why mutations in specific genes may result in isolated cleft lip, isolated cleft palate, or combined cleft lip and palate [26,27,28].

Understanding these developmental differences is critical for interpreting genetic findings and explains why cleft lip and cleft palate may occur independently or together in different individuals.

To facilitate understanding of the complex molecular mechanisms underlying craniofacial development, the major developmental events involved in lip and palate formation, together with their principal genes, transcription factors, signaling pathways, and representative references, are summarized in Table 1.

## 4. Genetic Classification and Major Genes in CL/P Pathogenesis

CL/P comprise a genetically heterogeneous group of craniofacial anomalies with diverse etiological mechanisms. From a genetic perspective, CL/P are broadly classified into syndromic and nonsyndromic forms based on the presence or absence of additional congenital anomalies and their underlying genetic architecture. This classification is essential for understanding disease pathogenesis, guiding genetic testing strategies, and informing recurrence risk assessment [5,27,28,29,30].

Although syndromic and nonsyndromic CL/P are discussed separately for clinical purposes, they should not be considered genetically independent disorders. Increasing evidence indicates that these conditions exist along a molecular continuum, with several genes contributing to both phenotypes depending on variant type, genetic background, and environmental influences. Accordingly, the present review emphasizes genes with the strongest evidence rather than providing an exhaustive catalogue of all reported susceptibility loci [5,31,32,33,34,35,36,37].

### 4.1. Syndromic Cleft Lip and Palate (SCL/P)

Syndromic cleft lip and palate (CL/P) accounts for approximately 30% of all cleft cases and occurs as part of a recognized genetic syndrome involving additional congenital anomalies. Although hundreds of syndromes have been associated with CL/P, a limited number of genes account for a substantial proportion of genetically characterized cases. Among these, *IRF6*, *TP63*, *TBX22*, *MSX1*, *CDH1*, and *SATB2* represent the best-established genes and have been extensively validated through genetic and functional studies.

*IRF6* is the most extensively studied gene in syndromic CL/P and is responsible for Van der Woude syndrome and popliteal pterygium syndrome, the most common syndromic forms of clefting. *IRF6* regulates epithelial differentiation and periderm formation, both of which are essential for normal fusion of the facial prominences. Mutations in this gene disrupt epithelial development and markedly increase the risk of cleft formation [17,37,38].

TP63 is another key regulator of craniofacial development and epithelial morphogenesis. Pathogenic variants cause several ectodermal dysplasia syndromes associated with cleft lip and palate by disrupting epithelial proliferation, differentiation, and tissue integrity [29,39,40,41,42,43].

Mutations in *TBX22* are the principal cause of X-linked cleft palate with ankyloglossia. *TBX22* plays an essential role in posterior palate development, and loss of function impairs palatal shelf growth and fusion [30,44].

Other well-established genes implicated in syndromic CL/P include *MSX1*, which regulates craniofacial patterning and odontogenesis, *CDH1*, which is involved in cell adhesion and epithelial integrity, and *SATB2*, which contributes to craniofacial skeletal development and palate formation. Mutations in these genes have been associated with a range of syndromic phenotypes involving cleft lip and palate.

Additional genes, including *PHF8*, *ESCO2*, *KMT2D*, and several others, have also been implicated in rarer syndromic forms of CL/P. However, the genes discussed above represent the major contributors and have the strongest genetic and functional evidence supporting their role in syndromic cleft pathogenesis [45,46,47,48].

### 4.2. Nonsyndromic Cleft Lip and Palate (NSCL/P)

Nonsyndromic cleft lip and palate (NSCL/P) accounts for approximately 70% of all cleft cases and results from complex interactions between multiple genetic variants and environmental factors. Genome-wide association studies (*GWAS*), linkage analyses, and sequencing studies have identified numerous susceptibility loci; however, only a subset of genes has been consistently replicated across different populations. The strongest evidence supports the involvement of *IRF6*, *ARHGAP29*, *VAX1*, *MAFB*, *PAX7*, *FOXE1*, *GRHL3*, *NTN1*, and *WNT3/WNT9B*, all of which play critical roles in craniofacial development and palatogenesis [49,50,51,52,53,54,55,56,57,58].

Although more than 50 susceptibility loci have been identified, many have modest effect sizes or population-specific associations. Ongoing *GWAS* and next-generation sequencing studies continue to identify additional rare variants and regulatory elements, further refining the genetic architecture of NSCL/P [49,50,51,52,53,54,55,56,57,58,59,60,61,62,63].

The major genes implicated in syndromic and nonsyndromic CL/P and their principal biological functions are summarized in Table 2.

Among these genes, *IRF6*, *TP63*, and *TBX22* are the most extensively characterized causes of syndromic CL/P, whereas *IRF6*, *ARHGAP29*, *VAX1*, and *MAFB* represent some of the strongest susceptibility genes for nonsyndromic CL/P.

### 4.3. Genetic Overlap and Clinical Implications

Although traditionally classified as distinct entities, syndromic and nonsyndromic CL/P share considerable genetic overlap. Genes such as *IRF6* and *TP63* are implicated in both forms, depending on the nature and functional impact of the variant. This supports a continuum model in which rare, high-impact mutations result in syndromic presentations, while common regulatory variants contribute to nonsyndromic susceptibility [38,39]. Furthermore, variable expressivity and incomplete penetrance observed in several CL/P-associated genes indicate that identical genetic variants may produce diverse clinical phenotypes, reflecting the influence of modifier genes, epigenetic regulation, and environmental factors.

This classification has important clinical implications. Syndromic cases typically require comprehensive genetic evaluation, including chromosomal microarray analysis, targeted gene panels, whole-exome sequencing, or whole-genome sequencing, whereas nonsyndromic cases are primarily managed through recurrence risk assessment and genetic counseling [40]. Advances in genomic technologies have improved the diagnostic yield, particularly in patients with atypical clinical features or multiple congenital anomalies, facilitating more accurate molecular diagnosis and individualized patient management.

Accurate classification also informs recurrence risk. Syndromic forms may carry significantly higher recurrence risks depending on inheritance patterns, while nonsyndromic CL/P generally presents lower but variable recurrence risks influenced by family history, disease severity, and the cumulative contribution of multiple genetic susceptibility loci [41]. Recognition of the genetic overlap between syndromic and nonsyndromic CL/P emphasizes the importance of integrating molecular findings with detailed clinical phenotyping to improve diagnosis, prognosis, genetic counseling, and future precision medicine approaches.

## 5. Molecular and Cellular Mechanisms Underlying Cleft Lip and Palate

Understanding how genetic susceptibility and environmental exposures converge at the molecular level is fundamental for elucidating the pathogenesis of cleft lip and/or palate (CL/P). Craniofacial development is a highly coordinated embryological process that depends on precise epithelial–mesenchymal interactions, governed by tightly regulated gene expression programs and interdependent signaling networks. Even subtle perturbations in these systems during narrow developmental windows can result in failure of facial prominence fusion and subsequent orofacial clefting.

### 5.1. Neural Crest Cell Development and Migration

Cranial neural crest cells (CNCCs) are multipotent, migratory progenitor cells that arise from the dorsal neural tube and contribute extensively to craniofacial morphogenesis. These cells form the mesenchymal core of the facial prominences and palatal shelves and give rise to a wide range of structures, including craniofacial cartilage and bone, connective tissue, and components of the peripheral nervous system. As such, CNCCs are indispensable for normal lip and palate formation.

The developmental trajectory of CNCCs involves sequential processes including specification, epithelial-to-mesenchymal transition (EMT), delamination, migration, proliferation, and lineage differentiation. Disruption at any stage of this tightly regulated cascade can result in abnormal craniofacial patterning and cleft formation [24]. CNCC migration is particularly sensitive to both genetic and environmental perturbations, as it requires coordinated cytoskeletal dynamics, cell–cell signaling, and extracellular matrix interactions.

Genetic studies have identified multiple key regulators of CNCC function implicated in CL/P. Genes such as *MSX1*, *PAX9*, and *SOX9* are expressed in neural crest-derived mesenchyme and play essential roles in craniofacial patterning, odontogenesis, and skeletal development. Loss or dysregulation of these genes disrupts morphogenetic signaling gradients and compromises facial prominence growth, thereby increasing susceptibility to clefting [25].

Additional transcription factors, including *PAX7*, *TFAP2A*, and *ZIC2*, are critical for early neural crest specification and survival. These factors regulate downstream gene networks involved in migration and apoptosis resistance during craniofacial development [64,65,66,67]. Importantly, CNCC development is highly sensitive to environmental influences. Maternal nutritional deficiencies (e.g., folate deficiency), oxidative stress, and teratogenic exposures (such as alcohol or certain medications) can alter epigenetic regulation and signaling pathways, exacerbating genetically predisposed defects. These combined disruptions ultimately impair CNCC contribution to facial prominences, reinforcing their central role in CL/P pathogenesis.

### 5.2. Epithelial Differentiation and Periderm Function

Successful fusion of facial prominences requires precise regulation of epithelial integrity, adhesion dynamics, and programmed cell removal. A critical transient structure in this process is the periderm, a superficial epithelial layer that prevents premature epithelial adhesion between developing facial structures.

The periderm is regulated by key developmental genes, including *IRF6*, *TP63*, and *GRHL3*, which collectively maintain epithelial homeostasis and prevent aberrant fusion events. Proper periderm differentiation ensures that opposing epithelial surfaces remain non-adherent until the appropriate fusion stage. Failure of periderm formation or timely desquamation results in persistent epithelial adhesions, mechanical obstruction of fusion, and ultimately cleft formation [61,62,63,64,65,66].

At the cellular level, palatal fusion requires coordinated removal of the medial edge epithelium (MEE), achieved through apoptosis, epithelial cell migration, and epithelial–mesenchymal transition (EMT). Disruption in any of these processes can lead to persistence of the epithelial seam, preventing mesenchymal continuity.

Recent integrative genomic studies combining human sequencing data with animal models have further demonstrated that many nonsyndromic cleft lip/palate (NSCL/P)-associated variants are located in regulatory enhancer regions. These elements exhibit temporally and spatially restricted activity during craniofacial development, suggesting that CL/P risk is frequently mediated through subtle dysregulation of gene expression rather than complete loss of gene function.

### 5.3. Signaling Pathways in Palatal Fusion

Palatal fusion is governed by an intricate network of signaling pathways that regulate epithelial–mesenchymal interactions, cellular proliferation, apoptosis, polarity, and extracellular matrix remodeling. The principal pathways involved include transforming growth factor-beta (TGF-β), Sonic hedgehog (SHH), bone morphogenetic protein (BMP), WNT, and fibroblast growth factor (FGF) signaling. Together, these pathways coordinate the growth, elevation, adhesion, and fusion of the palatal shelves [20]. Disruption of this signaling network represents a major etiological mechanism underlying cleft palate.

Among these, TGF-β signaling—particularly TGF-β3—is essential for palatal shelf fusion. TGF-β3 mediates the removal of the medial edge epithelium (MEE) by inducing apoptosis, regulating EMT, and promoting epithelial cell migration. This allows breakdown of the epithelial seam and subsequent mesenchymal continuity between opposing palatal shelves. Experimental knockout models have consistently demonstrated that loss of TGF-β3 function results in failure of MEE degradation and complete cleft palate formation [21]. In humans, variants in *TGFB3* have also been associated with increased susceptibility to nonsyndromic cleft palate, highlighting its translational relevance.

The SHH signaling pathway is essential for early craniofacial patterning and palatal shelf outgrowth. SHH regulates epithelial proliferation and mesenchymal expansion while maintaining reciprocal signaling interactions with FGF and BMP pathways. Disruption of SHH signaling leads to reduced palatal shelf size, midline defects, and abnormal craniofacial morphology, significantly increasing cleft risk [22].

BMP signaling contributes to mesenchymal proliferation, differentiation, and spatial patterning within the developing palate. It acts in a dose-dependent manner and is tightly modulated by antagonistic interactions with SHH and WNT pathways. Dysregulation of BMP signaling can impair tissue patterning and disrupt the coordinated growth required for shelf elevation and fusion [20,23].

The WNT signaling pathway plays a central role in cell fate specification, epithelial differentiation, and maintenance of tissue polarity. It regulates gene transcription programs essential for epithelial stability and morphogenesis. Aberrant WNT signaling disrupts epithelial adhesion and alters morphogenetic patterning, contributing to cleft formation [20,67].

FGF signaling regulates cellular proliferation, survival, and epithelial–mesenchymal communication during craniofacial development. It interacts closely with SHH signaling to ensure adequate palatal shelf growth and morphogenesis. Impairment of FGF signaling results in reduced mesenchymal expansion, delayed development, and failure of palatal shelf fusion [20,23].

Importantly, these signaling pathways function as an integrated regulatory network rather than independent cascades. Extensive crosstalk among TGF-β, SHH, BMP, WNT, and FGF pathways ensures precise temporal and spatial coordination of craniofacial development. Genetic mutations, epigenetic alterations, or environmental insults that disrupt this signaling equilibrium can lead to failure of palatal fusion and subsequent CL/P formation [20,67].

## 6. Conclusions

Cleft lip and palate (CL/P) are genetically and biologically heterogeneous craniofacial anomalies resulting from complex interactions between genetic susceptibility and environmental influences. Although syndromic and nonsyndromic CL/P are traditionally classified as distinct clinical entities, accumulating evidence indicates substantial genetic overlap between these forms, with several genes and developmental pathways contributing to both phenotypes depending on the nature and functional impact of the underlying genetic variants. This supports the concept of a molecular continuum rather than a strict genetic dichotomy.

Rather than providing an exhaustive catalogue of all reported susceptibility genes, this review focused on genes and molecular pathways supported by the strongest genetic, functional, and clinical evidence. By integrating findings from developmental biology, human genetics, genome-wide association studies, and gene–environment interaction research, this review highlights the key molecular mechanisms driving craniofacial development and cleft pathogenesis while emphasizing both the shared and distinct features of syndromic and nonsyndromic CL/P.

Continued advances in functional genomics, multi-omics technologies, and systems biology are expected to further clarify the roles of validated and newly identified susceptibility genes, improve genotype–phenotype correlations, and refine our understanding of shared molecular mechanisms underlying CL/P. These advances will facilitate more accurate risk assessment, genetic counseling, earlier diagnosis, and the development of precision medicine approaches for individuals affected by cleft lip and palate.

## Figures and Tables

**Table 1:** Major developmental events during lip and palate formation, the principal genes/transcription factors and signaling pathways involved, and representative references.

Developmental Event	Major Genes/Transcription Factors/Signaling Pathways	Representative References
Neural crest cell specification and migration	SOX9, TFAP2A, PAX7, WNT, BMP	[14,15]
Facial prominence growth	SHH, FGF8, BMP4	[7,8]
Primary palate formation	IRF6, TP63, GRHL3, TGF-β	[17]
Palatal shelf growth and elevation	SHH, FGF, BMP, TBX22	[18,19,20]
Medial edge epithelial seam disintegration	TGF-β3, IRF6, TP63	[19,20]
Secondary palate fusion	TGF-β3, WNT, BMP, FGF	[19,20]

**Table 2:** Major genes implicated in syndromic and nonsyndromic cleft lip and palate (CL/P), with their principal biological functions and associated clinical phenotypes.

Classification	Gene	Biological Function/Clinical Significance	Representative References
Syndromic CL/P	IRF6	Epithelial differentiation; Van der Woude syndrome; periderm formation	[17,40,41]
	TP63	Epithelial development; ectodermal dysplasia syndromes	[29,42,43]
	TBX22	Palatal development; X-linked cleft palate with ankyloglossia	[30,44]
	MSX1	Craniofacial morphogenesis and odontogenesis	[53]
	CDH1	Cell adhesion; hereditary diffuse gastric cancer syndrome with CL/P	[48]
	SATB2	Craniofacial skeletal and palate development	[59]
	GRHL3	Periderm formation; Van der Woude syndrome type 2	[40,41]
	KMT2D	Kabuki syndrome	[47]
	PHF8	X-linked intellectual disability with cleft lip/palate	[45]
	ESCO2	Roberts syndrome	[46]
Nonsyndromic CL/P	IRF6	Most consistently replicated susceptibility gene	[34,52]
	ARHGAP29	Craniofacial morphogenesis and palatal fusion	[59]
	VAX1	Facial patterning and morphogenesis	[49,56,57]
	MAFB	Neural crest cell differentiation	[49,56,57]
	PAX7	Neural crest specification and migration	[49,56,57]
	FOXE1	Craniofacial and thyroid development	[55]
	GRHL3	Epithelial differentiation and periderm integrity	[40,41]
	NTN1	Craniofacial development and tissue guidance	[59]
	WNT3	WNT signaling during facial development	[61,62,63,64,65,66]
	WNT9B	Palatal shelf growth and fusion	[61,62,63,64,65,66]
	BMP4	Cell proliferation and craniofacial patterning	[53,55]
	TGFB3	Medial edge epithelium disintegration and palatal fusion	[20,54,67]
	SHH	Palatal shelf growth and epithelial–mesenchymal signaling	[21]
	FGFR2	FGF signaling and tissue morphogenesis	[22]
	PAX9	Palatal and tooth development	[53]
	SOX9	Neural crest differentiation and chondrogenesis	[23,24]

## Data Availability

No new data were created or analyzed in this study.

## Abbreviations

The following abbreviations are used in this manuscript:

CL	Cleft Lip
CP	Cleft Palate
CL/P	Cleft Lip and Palate
NSCL/P	Nonsyndromic Cleft Lip and Palate
GWAS	Genome-Wide Association Study
IRF6	Interferon Regulatory Factor 6
TP63	Tumor Protein p63
TBX22	T-Box Transcription Factor 22
SHH	Sonic Hedgehog
BMP	Bone Morphogenetic Protein
FGF	Fibroblast Growth Factor
WNT	Wingless/Integrated
TGF-β	Transforming Growth Factor Beta
